# Assisted outpatient treatment: are court-ordered antipsychotic medications effective?

**DOI:** 10.1017/S1092852925000045

**Published:** 2025-01-28

**Authors:** Sophia Kocher, Marvin Swartz

**Affiliations:** 1 From the Wilson Center for Science and Justice, Duke University School of Law and the Duke School of Medicine; 2 The Department of Psychiatry and Behavioral Sciences, Duke School of Medicine and the Wilson Center for Science and Justice, Duke University School of Law, Duke University, Durham North Carolina

**Keywords:** Assisted outpatient treatment programs, Involuntary outpatient commitment, Antipsychotic medication, Medication effectiveness, Coercion, Ethical treatment

## Abstract

Assisted Outpatient Treatment (AOT) is a controversial civil court program wherein a judge orders a person with severe mental illness to adhere to an outpatient treatment plan designed to improve treatment adherence, prevent relapse and dangerous deterioration. Several states, including California and New York, have recently promoted use of AOT to try to address high rates of homelessness among person with severe mental illness. Under AOT, clinicians treating these patients must balance the ethical principles of patient autonomy and beneficence, and employ AOT only when previous treatment failed as a result of treatment non-adherence. However, some critics of AOT argue that not only is it coercive and ineffective but that the court mandate to adhere to prescribed medications, usually antipsychotic medications, compels AOT recipients to take ineffective and even harmful medications. This article examines the assertion of these critics and reviews the evidence of antipsychotic effectiveness and potential harms in treating psychotic disorders under a civil court order.

## Introduction

Assisted Outpatient Treatment (AOT) has emerged as a potential, but controversial response to treatment non-adherence, increased symptomatology such as severe hallucinations, and subsequent relapse for individuals with debilitating psychiatric illnesses.^1^ ^–^^6^ Several states, including California and New York, have recently promoted the use of AOT to try to address high rates of homelessness among persons with severe mental illness.

The American Psychiatric Association (APA) in its Position Statement on AOT^7^ describes AOT as “a civil court procedure wherein a judge orders a person with severe mental illness to adhere to an outpatient treatment plan designed to prevent relapse and dangerous deterioration.” It notes that the goal of such programs is to meet the needs of persons with severe mental illness whose complex treatment and human service needs are unmet by community mental health programs. Thus, these programs seek to reduce rates of relapse and hospitalization, the likelihood of dangerous behavior, and incarceration.^7^ In doing so, programs must balance the ethical principles of patient autonomy and beneficence, resorting to AOT only when previous treatment options fail. Per the APA, these programs also must be implemented in a nondiscriminatory manner to ensure they are fairly applied and respectful.^7^

## The controversies about AOT

Those in favor of AOT argue that it can promote access to treatment for those with severe mental illnesses such as schizophrenia, bipolar disorder, major depression, panic disorder, obsessive-compulsive disorder, and post-traumatic stress disorder.^1^ The court order not only commits a patient to receive treatment, it also commits the health system to provide that care. While AOT programs may encroach on patient autonomy, mandated outpatient regimens are far less restrictive than hospitalization, homelessness, or incarceration.^4^ In addition, proponents of AOT argue that, while AOT-type interventions limit autonomy in the short run, they can restore a more durable autonomy in returning patients to community independence and better functioning.^8^ ^,^^9^ As a corollary, they also argue that under the throes of a full-blown psychosis, a person controlled by delusions and hallucinations is not fully autonomous.^8^ ^,^^9^ In fact, repeated cycles of involuntary hospitalizations are one of the negative outcomes these programs aim to prevent. Proponents also argue that AOT serves as an earlier point of entry into care systems, including ones for social support, thus reducing prolonged social isolation and suffering. Critics focus on AOT’s coercive nature and the social/political/legal ramifications of framing medical non-adherence as a legal matter and subjecting patients to potential criminalization.^10^ Still, others criticize AOT programs for not being stringent enough to be effective.^1^ ^,^^5^ ^,^^6^

Central to these critiques is the question of whether AOT can actually “work.” Studies of AOT effectiveness have shown mixed results arguably as a result of its implementation.^1^ ^,^^5^ ^,^^6^ Several studies demonstrate positive outcomes, but studies have varied methodologies, inconsistent implementation, different outcome measures, and many lack generalizability. Further implementation of these programs is very situationally dependent, in that they are implemented in varied communities with varied resources and systems of care.^1^ ^,^^2^ ^,^^5^ ^,^^6^ For example, several studies conducted in the United States were compared to one in the United Kingdom, which are vastly different systems of care.^6^ What the literature *does* demonstrate is that the success of AOT depends on successful implementation, good treatment resources, and an adequate duration of court-ordered treatment.^6^ Therefore, when thinking about AOT in relation to other community-based interventions it may be time to shift the question. When caring for individuals with severe mental illness we should be asking: *for whom* does court-mandated outpatient treatment work and *under what conditions*?

The APA consensus on this question is that AOT can play a significant role in the recovery of individuals with severe mental illness when programs are well-planned, offer intensive and individualized services, and last for a sustained period of time.^7^ Centered around intensive outpatient services, these programs can enhance treatment adherence and reduce hospitalization rates; and for a subset of the patient population, they aim to mitigate the likelihood of dangerous behavior. However, AOT should not be seen as a primary tool for preventing violence.^5^ Instead, programs should seek to mobilize treatment resources with a focus on preventing patients’ severe deterioration.

Assessments of for whom these programs would most benefit should be based on past clinical history of relapse due to treatment non-adherence.^7^ AOT is especially vital to assist patients at risk of relapse who are unlikely to seek treatment voluntarily due to their mental illness. In terms of what services programs should offer, the APA cites research showing that comprehensive services, including medication management and psychosocial support, enhance AOT’s effectiveness.^6^ The APA also recommends thorough psychiatric and physical examinations to address co-occurring medical issues^7^ Of note, although psychotropic medication often plays a crucial role in the treatment plan for patients, involuntary administration of medications is not authorized under AOT and requires separate legal authority and approval. That is, patients under AOT are ordered by the court to comply with treatment recommendations but they cannot be forcibly administered medication and legal charges cannot be levied against non-adherent patients. Indeed, the legal sanction for non-adherence is a law enforcement transport for an examination to assess whether a higher level of care is needed.

The APA further recommends that AOT programs be held accountable for implementation in maximizing the success of the court orders.^7^ Key factors in success are clinician involvement in treatment planning and engaging patients and families in treatment preferences whenever possible. Even under court-ordered treatment, the APA recommends close collaboration with the patient to select medication regimens that are tolerated and effective.^7^ In addition, the APA stipulates that patients should be provided due process legal protections, similar to those afforded for involuntary hospitalization. Finally, regular evaluations of programs should be conducted to ensure equitable application and to address any disproportionate use among minority groups.

Some critics of AOT argue that not only is AOT coercive and ineffective but that the court mandate to adhere to prescribed medications, most often antipsychotic medications, compels AOT recipients to take ineffective and harmful medications. As one opponent said:

What would you want if you were in this position? Do you want to be forced to take a medication that you feel has really harmful side effects? I want to change the narrative on this and make it about choice.

(Accessed October 30, 2024. https://www.madinamerica.com/2024/05/maryland-enacts-a-draconian-assisted-outpatient-treatment-program/).

## Are antipsychotics effective and tolerable?

The treatment of acute schizophrenia and other psychotic disorders does present challenges, one major one being patient adherence to prescribed therapies. Utilizing the court order, AOT can be a potentially effective strategy to ensure consistent adherence to antipsychotic medications. However, mandating antipsychotic treatment is criticized by some physicians, patients, members of the public, and policymakers due to their known side effects and limited effectiveness–suggesting that their use under court order be minimized. The following brief review considers the effectiveness and side effects of antipsychotic medications, informing discussions about their use as part of AOT.

A systematic meta-analysis by Leucht et al. examines the effectiveness of antipsychotic drugs for the treatment of acute exacerbations of schizophrenia compared to placebo responses in clinical trials over the past 60 years.^11^ Leucht et al. analyzed 167 double-blind randomized controlled trials, totaling 28 102, mainly chronic, patients. Their work highlights the efficacy of antipsychotic medications, revealing that approximately twice as many patients improved with antipsychotics compared to placebo. They found that 51% of the antipsychotic group experienced at least a “minimal” symptom response, defined as either at least a 20% reduction from baseline on common symptom rating scales (e.g., the PANSS, BPRS, or Clinical Global Impression Scale) indicating at least “slightly improved” or better, compared to 30% response in the placebo group. Still, only a minority experienced a “good” response, though the majority of those who were improved were in the antipsychotic group.^11^ These data support arguments that antipsychotic medication as part of the treatment regimen under AOT can enhance treatment effectiveness.

However, the review also reveals that while antipsychotics can be effective, they often have significant side effects. In their meta-analysis, antipsychotic drugs were associated with more movement disorders, more sedation, more weight gain, prolactin increases, and more electrocardiogram (EKG) QT interval prolongation than placebo. In their analysis, effect sizes of side effect differences across different drug types demonstrated significant heterogeneity, reflecting the differences in individual antipsychotics. This difference in side effect profiles was most notable between first (older) and second (newer) generation antipsychotics as both classes were included in the meta-analysis.^11^ This variation presents an opportunity for intervention: careful drug choices and modifications of individualized treatment regimens can reduce side effect burdens for patients. The structured programing and prolonged treatment courses of AOT provide a chance for providers to find an efficacious treatment regimen while selecting a specific medication to minimize side effects and promote the best possible outcomes. It is important to note that even when prescribing under court oversight the physician’s ethical duty continues to be to the patient, meaning he/she has a duty to find a treatment plan that is tolerable and effective. AOT does not absolve the physician of his/her fidelity to good ethical care.

In their review, Leucht et al. further respond to questions about the efficacy of antipsychotics.^11^ Skepticism about whether these drugs actually “work” likely stems from a number of trials in recent years that show older drugs, like haloperidol, failing to outperform placebo. Their meta-analysis identifies an increased placebo response over time as a moderator of drug efficacy, rather than a decrease in drug response itself.^11^ This suggests that the apparent decrease in drug efficacy in trials may well be influenced more by improved patient response to placebo rather than a true decline in the effectiveness of the drugs per se. They also note that trends of decreasing effect size (superiority of drug over placebo) in clinical trials of recent years may be an artifact of study design. More recent studies tend to use standardized criteria for assessment of improvement as well as larger and more diverse samples, both of which can decrease effect sizes.^11^ Thus, this trend should not be thought of solely as antipsychotic drugs becoming less efficacious.

In response to potential sources of bias and the perceived integrity of drug treatment, Leucht et al. discuss the significant impact of industry sponsorship on effect sizes.^11^ Surprisingly, they found industry sponsorship of drug trials to be associated with smaller effect sizes, which they hypothesize is due to large studies that involve multiple countries and study sites with different populations and multiple raters administrating rating scales. Multiple raters lead to increasing variability in rating scores and increased measurement error. AOT may provide a structured treatment framework that may help reveal the true benefits of these medications in a real-world setting, offering insights that are less influenced by the placebo effects.

Finally, and perhaps most importantly, Leucht’s review highlights the improvement of quality of life and social functioning with antipsychotic treatment, even in the short term (6 weeks). AOT has the potential to enhance these benefits by promoting adherence and continuity of care, supporting individuals with severe mental illness in achieving better health outcomes.

Antipsychotics are also the mainstay treatment for long-term maintenance treatment for patients with schizophrenia. In their comprehensive Cochrane review, Ceraso et al. examine whether antipsychotics are effective for relapse prevention, in addition to their ability to reduce acute symptoms of schizophrenia.^12^ They reviewed 75 randomized controlled trials involving 9145 participants to see the effects of maintenance medication compared to stopping antipsychotic agents.

They found that antipsychotics significantly reduced the risk of relapse compared to placebo. This finding was consistent across studies and time frames. Indeed, risk ratios suggest that the likelihood of relapse was nearly three times higher (RR 0.38) for those not on medication maintenance treatment (at 7–12 months). Those in the placebo group were more likely to leave the study early, both for any cause and due to the inefficacy of the intervention. Antipsychotic use was also associated with decreased hospitalization. Importantly, these effects persisted even when accounting for participants who had been stable for various periods (1–3 months) before the start of a trial.^12^ Antipsychotic medication uses still reduced relapse rates (with no difference between the duration of pre-trial stability), demonstrating the robust sustained efficacy of antipsychotics in preventing symptom recurrence over time.

Further, these studies demonstrate that quality of life may be superior for patients treated with antipsychotics. Although fewer studies measured this outcome, there was a clear and statistically significant improvement in quality-of-life measurements for those taking antipsychotics. In their pooled data, they found substantial heterogeneity in the amount of improvement, due in part to the use of different scales across studies, yet all had the same trend toward improvement. Also in their analysis, they found those in the antipsychotic group reported improved social functioning.^12^ Medication management had a positive effect on the ability to engage in activities and relationships– they found that those continuing treatment tended to experience higher satisfaction with their life and with their treatment. Of key importance, poor adherence to prescribed antipsychotics and repeated episodes of psychoses clearly impact long-term outcomes of schizophrenia including poorer functioning, higher risk of hospitalization, arrest, violence, victimization, poorer life satisfaction, and greater substance use, and alcohol-related problems.^13^ These improvements must be weighed against the side effects of medication therapy. The review found antipsychotic medications, in the long term (>3 months), associated with a greater number of movement disorders (e.g., akathisia, akinesia, dyskinesia, dystonia, tremor), and increased weight gain and sedation.^12^ The review was also limited by the duration of follow-up. Studies included in the analyses generally lasted up to 1 year, meaning that further work must be done to assess the long-term morbidity and mortality of these drugs as well as the potential impact of social/environmental factors on remission of symptoms with antipsychotic treatment. Nevertheless, the authors conclude that “stopping treatment [may be] far more harmful than thoughtfully maintaining it.^12^

With regard to the side effects of antipsychotics, the types of adverse effects are diverse. In their systematic review, Young et al. reveal that side effects are common and that their incidence increases with antipsychotic polypharmacy and increased duration.^12^ Moreover, different drugs were found to have different side effect profiles. For example, clozapine was more strongly associated with metabolic disturbance and olanzapine was associated with the most weight gain.

Importantly, these side effects can be managed. However, Young et al. found that despite clinical guideline recommendations, there was a disappointing rate of baseline monitoring and follow-up: for lipid monitoring, glucose monitoring, and no evidence of evaluation for sexual dysfunction (the side effect they found to be most common).^14^ Because antipsychotic drugs vary in their side effects, increased monitoring would allow for interventions to better manage side effects. Of the studies they reviewed, the ones that assessed the efficacy of adverse effect management strategies found effective non-therapeutic and therapeutic interventions that resulted in improved control of adverse effects. For example, one found significant decreases in weight gain with a program of physical exercise, diet therapy, and group therapy.^15^ Another found a significant decrease in cholesterol and triglycerides with statin therapy.^16^ Still, Young et al. note that only a minority of patients receive these interventions. One of the studies in their review found that few patients were receiving lipid-lowering therapy and only a minority received antihypertensive medications.^17^ ^,^ ^18^ These findings underscore a need for greater emphasis on managing antipsychotic side effects. This also suggests that courts could play an important role in emphasizing standards of care in prescribing medications.

These reviews underscore that antipsychotic drugs are indeed demonstrably effective in reducing relapse, improving symptoms and reducing hospitalization. However, antipsychotic drugs do increase the risk of troublesome side effects, including health risks such as weight gain. This puts the responsibility on the physician treating an AOT patient to prescribe wisely and to find the most tolerable medication regimens that reduce risk and maximize benefit. In many ways, this is no different than the role of the physician working with a “voluntary” patient. In either case, treatment adherence is largely driven by collaboratively finding a regimen that is acceptable and tolerable to the patient. And importantly, professional medical ethics demand that the physician honor the physician-patient relationship by finding a treatment regimen that the patient can best tolerate.^18^

## Conclusion

These reviews provide solid evidence that antipsychotic regimens are demonstrably effective in treating psychotic symptoms and that maintenance antipsychotic treatment reduces relapse and poor outcomes. Antipsychotics are the consensus treatment of choice for patients with psychotic disorders. These reviews also make clear that there is a substantial side effect burden associated with these medications. Absent careful monitoring and side effect management, certain side effects can have serious long-term effects. As a result, it is incumbent on the treating clinician to monitor side effects carefully and make changes in the medication regimen to find the most tolerable regimen. If AOT required a single fixed and unchanging regimen of medications it would clearly pose an ethical problem for the treating clinicians and be unfair to the patients under AOT. However, that is not the case, AOT clinicians can and are ethically obliged to to collaborate with AOT patients to find tolerable and effective medication regimens.
